# Improving* In Vivo* Efficacy of Bioactive Molecules: An Overview of Potentially Antitumor Phytochemicals and Currently Available Lipid-Based Delivery Systems

**DOI:** 10.1155/2017/7351976

**Published:** 2017-05-07

**Authors:** Lamia Mouhid, Marta Corzo-Martínez, Carlos Torres, Luis Vázquez, Guillermo Reglero, Tiziana Fornari, Ana Ramírez de Molina

**Affiliations:** ^1^Molecular Oncology and Nutritional Genomics of Cancer, IMDEA Food Institute, CEI UAM+CSIC, Madrid, Spain; ^2^Department of Production and Characterization of Novel Foods, Institute of Food Science Research (CIAL), Campus of International Excellence (CEI) UAM+CSIC, 28049 Madrid, Spain

## Abstract

Cancer is among the leading causes of morbidity and mortality worldwide. Many of the chemotherapeutic agents used in cancer treatment exhibit cell toxicity and display teratogenic effect on nontumor cells. Therefore, the search for alternative compounds which are effective against tumor cells but reduce toxicity against nontumor ones is of great importance in the progress or development of cancer treatments. In this sense, scientific knowledge about relevant aspects of nutrition intimately involved in the development and progression of cancer progresses rapidly. Phytochemicals, considered as bioactive ingredients present in plant products, have shown promising effects as potential therapeutic/preventive agents on cancer in several* in vitro* and* in vivo* assays. However, despite their bioactive properties, phytochemicals are still not commonly used in clinical practice due to several reasons, mainly attributed to their poor bioavailability. In this sense, new formulation strategies are proposed as carriers to improve their bioefficacy, highlighting the use of lipid-based delivery systems. Here, we review the potential antitumoral activity of the bioactive compounds derived from plants and the current studies carried out in animal and human models. Furthermore, their association with lipids as a formulation strategy to enhance their efficacy* in vivo* is also reported. The development of high effective bioactive supplements for cancer treatment based on the improvement of their bioavailability goes through this association.

## 1. Introduction

The conventional treatments against cancer are nowadays replaced by new approaches such as hormone therapy, biological therapy, and stem cell transplantation. In addition to these proposals, new chemical compounds are tested, focusing on founding antitumoral agents with high specificity response and low toxic side effects and warding off resistance development. In this sense, phytochemicals (Phy) have received increasing attention due to their high potency and low toxicity compared with common chemotherapeutic agents [1] and with pharmacological properties acting through specific molecular targets [2–4]. Thus, Phy are considered as nonnutritive compounds found in plants and safe for human intake [2] and with promising applications since their consumption is integrated within diet components.

However, despite their promising benefits* in vitro*, results from several studies highlight a low Phy bioactivity* in vivo* [5], mainly attributed to their poor water solubility, rapid metabolism, and short half-live and even causing gastrointestinal irritation. These factors lead to low and variable oral bioavailability and nonreproducible absorption, which gives rise to therapeutic concentrations that are difficult to achieve, high intra- and intersubject variability, and lack of dose proportionality [6], offering significant limitations or challenges to the cancer therapy with Phy.

Therefore, to increase the Phy applicability, developing formulation strategies that overcome limited oral bioavailability of Phy is needed. In this sense, the association of Phy to delivery systems or carriers composed of diverse materials has been proposed [7]. Particularly, in the last decade, association with lipids, usually referred to as lipid-based delivery systems, gained much interest as they are nontoxic, biodegradable, and highly biocompatible and show great versatility. In this respect, lipid formulations can be modified in various ways to meet a wide range of product stability requirements (molecular weight and physicochemical properties), disease conditions and route of administration, and existing commercial formulations for topical, oral, pulmonary, or parenteral product delivery [8, 9].

In these frameworks, the present work summarizes the existing dietary Phy with promising anticarcinogenic properties and Phy-based therapies that are being currently evaluated* in vitro*,* in vivo*, and in clinical trials as efficient approaches for the prevention and treatment of cancer and their bioavailability. Likewise, it also summarizes the delivery systems currently used to enhance the clinical use of Phy by increasing their oral bioavailability and by promoting their safe and targeted activity, mainly emphasizing the lipid-based delivery systems.

## 2. Dietary Phytochemicals Possessing Anticancer Properties

In the last years, several studies have amply demonstrated that tumor development could be highly associated with diet habits [10, 11]. In this sense, current researches on new approaches for cancer treatment are focused on the study of three axes: dietary patterns, specific foods, and safe and bioavailable dietary compounds [12]. Among the latter, Phy derived from diet might be considered as promising preventive and therapeutic alternative agents against cancer.

According to their chemical structure, Phy can be mainly classified into four groups: polyphenols, terpenes, organosulfur compounds, and phytosterols. The following provides a description of Phy belonging to the mentioned structural categories that have shown potential anticancer properties in* in vitro* studies, as the first step to evaluate their enhanced activities, and in* in vivo* models, as the second step of efficacy evaluation and determination of molecular action and targets. Phy tested in preclinical and clinical studies conducted with human cancer patients to validate their* in vivo* therapeutic effect are also listed.

### 2.1. Polyphenols

Antitumor benefits of polyphenols have been widely described. Polyphenols constitute one of the major constituents of plants and are abundant in our diet. The occurrence in plant matrix is very variable, going from simple phenolic molecules to complex associations (highly polymerized compounds). They are usually classified into different groups according to their structure and number of rings, highlighting phenolic acids, flavonoids, stilbenes, and curcuminoids, which are described below and compiled in Table 1.

(i)* Phenolic acids* represent 30% of total dietary polyphenols [13] and they are the major constituents of phenolic compounds. They usually include hydroxybenzoic acids and hydroxycinnamic acids [14], where one of the positions of the aromatic benzoic o cinnamic ring is occupied by a hydroxyl group and the remaining four positions are available for other chemical groups. One of the most studied phenolic compounds is the ellagic acid, as described in Table 1.

(ii)* Flavonoids*. Although they are not considered as essential dietary factors, they represent 60% of dietary polyphenols [2, 13] and are starting to be considered the key between prevention and treatment of chronical diseases and diet. Chemically, the flavonoid skeleton consists of two phenyl rings joined by a linear three-carbon bridge [15]. Table 1 summarizes those studied against cancer. Genistein, (−)-epigallocatechin-3-gallate (EGCG), and quercetin are the flavonoids more frequently tested in clinical trials against tumors. Genistein have been extensively studied as prospective antitumor molecules in the treatment of prostate cancer. Meanwhile, EGCG has also been largely studied in experimental studies against different types of tumors, even in clinical trials, particularly against prostate or cervical injuries. Quercetin was tested, in addition, against tumors related to the digestive tract, such as bowel, colon, or pancreas.

Within flavonoids, proanthocyanidins are also underlined as effective naturally occurring compounds in grape seeds or pine bark with antitumorigenic effects. They take the form of oligomers or polymers (+) catechin and (−) epicatechin, and the carried-out* in vivo* studies have remarked the preventive and effective action against UV-induced skin tumors but also showed the inhibition of lung metastasis and mammary and prostate cancer [16]. Concerning clinical studies, the is just one concluded trial which studied the positive chemoprevention proanthocyanidin effect on breast cancer [17].

(iii)* Stilbenes* constitute a large family within polyphenols and have numerous implications in plant disease resistance and human health (including antitumoral activity). Stilbenes have a 1,2-diphenylethylene core and belong to a small group of phenylpropanoids and only a few plants spices can synthetize them. They are produced in response to a biotic or abiotic stress [18]. The most largely studied is resveratrol, which is produced in plants in response to mechanical injuries. It is reported to be efficient against gastrointestinal tumors in clinical trials, and* in vivo* tests were carried out in breast, ovarian, lung, or skin tumors (Table 1).

(iv)* Curcuminoids* are derived from curcumin, and they are obtained from turmeric* (Curcuma longa)*. Curcumin belongs to diarylheptanoid series and is characterized by 1,3-diketones and two methoxylated phenols [19]. Curcumin is largely used as medicinal and food ingredient in Asia, especially in India. Within cancer therapies, it has been tested in several* in vivo* tumor models and even in clinical trials (Table 1).

### 2.2. Terpenes

Another important group of phytochemicals is that constituted by terpenoids or terpenes, which is the most abundant and structurally diverse group synthetized by plants. Terpenes show a wide range of physiological functions, many of them related to the plant defense system, and they are often components of essential oils and resins [20]. Terpenes are synthesized from two to five carbon building blocks based upon the isoprene unit. Depending on the number of blocks, they can be classified as monoterpenes (C10), sesquiterpenes (C15), diterpenes (C20), triterpenes (C30), tetraterpenes (C40), and polyterpenes [21]. Their potential antitumor properties have been described in several works [22], as shown in Table 2.

(i)* Carotenoids* are the most abundant tetraterpenes, and in natural samples they could be found free or esterified by fatty acids, the degree of esterification being related to the hydroxyl groups. They also are characterized by the presence of 11 or 12 conjugated carbon double bounds [23]. All of them represent variants or degradation derivatives of *β*-carotene, which is found in carrot* (Daucus carota)*. Antitumor activity of the acyclic tetraterpene lycopene has been largely studied in both* in vivo* and clinical trials, especially conducted with prostate tumors (Table 2). Besides lycopene, astaxanthin may exert antitumor activity through its antioxidant and immunomodulatory characteristics in tumors such as colon and hepatic carcinomas, as shown in Table 2.

(ii)* Noncarotenoids* are not derived from carotenes. This group of terpenes includes carnosol, a phenolic diterpene largely studied in cancer and associated with bioactivity of rosemary (Table 2). For carnosol, there are* in vivo* positive studies against colon, prostate, and skin tumors and no clinical studies proposed.

### 2.3. Organosulfur Compounds

Organosulfur compounds are Phy with one or more carbon-sulfur bonds in their structure and a thioketal-linked glucose molecule (S-glycosides). They are classified into two groups: glucosinolates and thiosulfinates [24]. Glucosinolates are sulfur-containing plant secondary metabolites that usually exist in cruciferous plants and are hydrolyzed by specific enzymes (myrosinases) to release biologically active sulfurated aglycones, known as isothiocyanates [2, 25]. Glucosinolates and their hydrolysis products exhibit direct and indirect antioxidant effects by scavenging harmful radicals and modulation of detoxification enzymes, such as glutathione S-transferase [26]. Thus, consumption of cruciferous plants, such as cabbage and broccoli, is believed to promote health and to reduce the risk of cancer development [27]. Among isothiocyanates, sulforaphane, produced from the glucosinolate glucoraphanin, has been largely studied as chemopreventive agent in different tumors* in vivo*, and it is the unique organosulfur compound that has been tested in a clinical trial as antitumorigenic agent [28] (Table 2).

Thiosulfinates (allyl sulfides), such as diallyl sulfide (DAS), diallyl disulfide (DADS), and diallyl trisulfide (DATS), are mainly present in garlic and onion (Allium family) [25]. Among them, DADS, an oil-soluble organosulfur compound, has been described as the major one responsible for therapeutic properties against prostate and colon in* in vitro* models and gastric, breast, and leukemia in* in vivo* models (Table 2).

### 2.4. Phytosterols

Phytosterols are lipid-like compounds and essential for maintaining permeability and fluidity on cell plant permeability. Vegetable oils are the main source of dietary phytosterols. They occur in various structural forms (as steryl glucosides, acetylated steryl glucosides, esters, or alcohols) [29], each of them existing in different compartments of the plant cell. There are approximately 200 phytosterols, among which *β*-sitosterol, campesterol, and sitostanol are the major ones [30].

*β*-Sitosterol is the most abundant phytosterol and although it is well known for its cholesterol lowering action [31], several* in vitro* and* in vivo* evidences suggest it possesses preventive effects against cancer (Table 2). Campesterol and sitostanol, however, have not shown any effect on tumor growth [32].

Within terpenes, triterpenoids (squalene) play a determinant role as they are considered common precursors of steroids, including phytosterols. Triterpenoids exist in free form or combined with sugar into glycosides. The free form shares the same chemical properties as phytosterol so long as they can be dissolved in organic solvents but insoluble in water [33]. In the last years, triterpenoids have demonstrated antitumor efficacy against breast, leukemia, multiple myeloma, and non-small cell lung carcinomas, specially affecting cell proliferation [34, 35]. Some triterpenes are already tested in Phase I clinical trials [36], with beneficial effects, even if some authors defend their combination with other triterpenoids, Phy, or synthetic drugs.

In general,* in vitro* and* in vivo* assays conducted with dietary Phy (Tables 1 and 2) showed tumorigenesis inhibition or potential chemopreventive effects. However, a high variability in anticancer effects was observed among different patients during clinical trials, which is one of the major limitations of the Phy-based therapy in the clinical practice.

## 3. *In Vivo* and Clinical Bioactivity of Phytochemicals

Although Phy hold part of their biological activity* in vivo*, as said above, their activity in this context is lower than observed for the same compound in the* in vitro* evaluation phase. An obvious reason for the “loss” of activity is the lack of pharmacokinetic optimization or compatibility [37]. One of the main factors that influences pharmacokinetics of the tested bioactive compound is its tissue bioavailability, which is defined by the Food and Drug Administration as* “the rate and extent to which the active ingredient or active moiety is absorbed from a drug product, reach plasma and body tissues and becomes available at the site of action in an unchanged form”*. Thus, bioavailability should be considered when the efficacy of dietary Phy is evaluated* in vivo* in animal models and/or human clinical trials. The impact of bioavailability is especially pronounced when the bioactive compound is intended for oral use, whereby gastrointestinal (GI) absorption constitutes the primary barrier between an active ingredient and systemic circulation. In the present review, we focus on oral bioavailability as the major pharmacokinetic aspect for the clinical application of orally delivered dietary Phy with high bioefficacy as anticancer agents. In this respect, factors affecting GI absorption and oral bioavailability of main dietary Phy will be addressed.

### 3.1. Oral Bioavailability of Dietary Phytochemicals

Oral route is generally considered the easiest and most convenient method for the delivery of drugs and dietary bioactive compounds due to properties such as noninvasiveness, cost-effectiveness, and being less prone to side effects, such as injection-site reactions [9]. In fact, although in some of the* in vivo* studies and clinical trials listed in Tables 1 and 2 Phy were administered by intraperitoneal or intratumoral injection and topical route [38–44], in most of the cases, they were orally administered (by gavage, diet supplementation, water suspension, or capsules).

**Figure 1:**
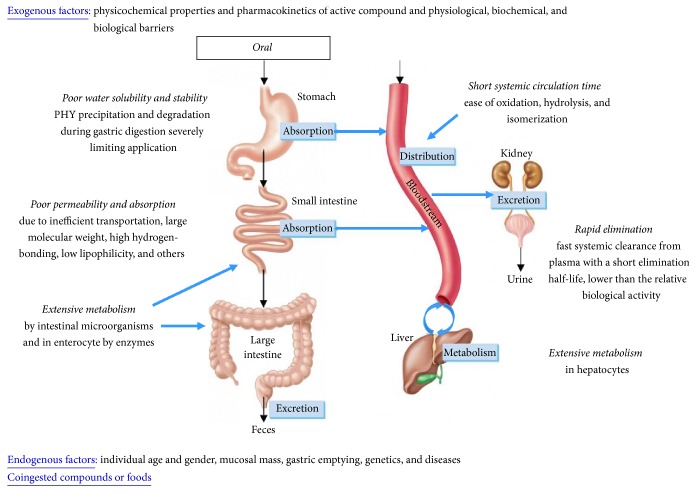
Determinant factors of the oral bioavailability of bioactive compounds, including phytochemicals.

However, as commented above, the suitability of this administration route depends on the oral bioavailability of the active ingredient, which, as summarized in Figure 1, is the result of the synergistic effect of the following factors:

- Physicochemical properties of Phy, which determine their water solubility and stability inside the GI tract
- Physiological barriers, including the chemical (e.g., pH) and biological environment (e.g., microbiota) inside the GI tract, which also have a significant influence on Phy stability during digestion and absorption [45]
- Biochemical barriers (including biodistribution), biological barrier (GI wall permeability), and pharmacokinetics (metabolism and clearance) of the active ingredient
- Endogenous factors, as the individual age and gender, mucosal mass, gastric emptying, genetics, and diseases [46]
- Amount of coingested compounds or foods

A compound which can exist in a stable form to survive the GI environment and that has optimum physicochemical properties to penetrate the GI wall is most likely to possess acceptable oral bioavailability. Most of Phy, however, have shown physicochemical properties that lead to a poor water solubility and stability in the GI environment and poor permeability. These include complex structure, size, high molecular weight, high lipophilicity, compound H-bonding to solvent, intramolecular H-bonding, intermolecular H-bonding, crystal packing, crystallinity, polymorphic forms, ionic charge status, isoelectric point (pI), and salt form [47]. In addition to physicochemical properties limiting their GI absorption, Phy are usually subjected to extensive metabolism in the enterocyte and hepatocyte and/or quickly eliminated in the urine [48]. All these factors result in a poor and variable bioavailability, which leads to therapeutic concentrations that are difficult to achieve, nonreproducible absorption, variable efficacy intra- and intersubject during clinical trials, and lack of dose proportionality. This explains the lower* in vivo* bioactivity and nonreproducible data obtained in previous studies (Table 2) [6, 49]. Bioavailability studies of the major dietary Phy are described below.

#### 3.1.1. Bioavailability Studies of the Major Dietary Phytochemicals

*(i) Polyphenols*. Most of the studies focus on bioavailability related to levels of the polyphenol present in blood or urine [50], but few of them determine the bioavailability in target tissues, which can be more determinant for affirming their application for a specific illness. After intestinal hydrolysis, polyphenols are conjugated by glucuronidation (addition of glucuronic acid), methylation (addition of a methyl group), or sulfurylation (addition of a sulfo-group), which often facilitate their urinary elimination. Thus, they are well absorbed on tissues where they are metabolized (bowel and liver) [51, 52], but their bioavailability in target tissues is low because of their rapid clearance from the body.

Nevertheless, there is a study that reveals that once sulfate and glucuronide conjugates of resveratrol are circulating in plasma (with an expected low bioavailability), their subsequent hydrolysis releases free resveratrol which can be captured by those cells with specific membrane receptors, increasing thus its bioavailability in specific tissues [53].

These conjugations may also depend on factors described in Section 3.1 such as age and gender, genetics and diseases, and protein-binding in tissues and blood. Moreover, independently of the mechanistic processing of flavonoids, some authors have also described the preventive efficacy of flavonoids (resveratrol) as dependent on the type of diet. In this sense, it has been demonstrated that low doses of resveratrol were able to reduce colon tumor progression better than high doses in subjects exposed to a high fat diet. [54].

*(ii) Terpenes*. Clinical relevancies of terpenes depend on their presence in target organs. Terpenes have a high lipophilic behavior, and therefore they depend on their solubility in the aqueous phase of the gut lumen. Thus, it has been observed that bioavailability of terpenes is related upon their incorporation to a lipid phase either during digestion or during food processing, making the presence of a quantity of fat necessary for their absorption [20]. Lycopene, one of the major carotenoids described for its anticarcinogenic potential, has been demonstrated to enhance its bioavailability when they are integrated in a chylomicron [55].

*(iii) Organosulfur Compounds*. Studies related to organosulfur compounds are frequently carried out in combination with other Phy or drugs. Indeed, few experimental data determine their bioavailability, and urine levels after uptake of Brussels or broccoli sprouts [56] are the unique parameter usually measured.

But as they are increasingly consumed due to their potential antitumoral effects, a new variety with genetic variations has been proposed increasing thus the expression of transcription factors involved in glucosinolate biosynthesis. The resulting broccoli could deliver a larger amount of glucoraphanin (active sulforaphane) in plasma and urine [57], although it has not been evaluated in specific organs levels.

*(iv) Phytosterols*. Phytosterol structure is similar to that of cholesterol but each phytosterol has an additional side chain, which confers dissimilarities in their absorption. Low bioavailability of phytosterols is reported in human plasma after intake. Before absorption starts, the esters are split in the duodenum, increasing their hydrophobicity and reducing their absorption at the same time. In addition, it has been described that they poorly reesterify in the enterocytes, explaining their poor absorption and their subsequently low concentration in the blood circulation [58, 59].

## 4. Use of Lipid-Based Delivery Systems to Increase the Clinical Efficacy of Antitumor Phytochemicals Administered Orally

The development of crystalline solid formulations by modifying physicochemical properties, as salt formation and micronization (particle size reduction), was initially adopted to amend the poor water solubility of Phy [60]. However, the low wettability and handling difficulties of reduced size formulations as well as the aggregation of nanocrystals inside the body and the impossibility of salt formation from neutral compounds limit the use of these approaches [61]. Amorphous formulations, including solid solutions (active compound immobilized in polymer) and self-dispersing solid solutions (with surfactants), have been also applied; however, the questionable physical stability of product (possibility of crystallization of drug or polymer) limited their use [62].

**Figure 2:**
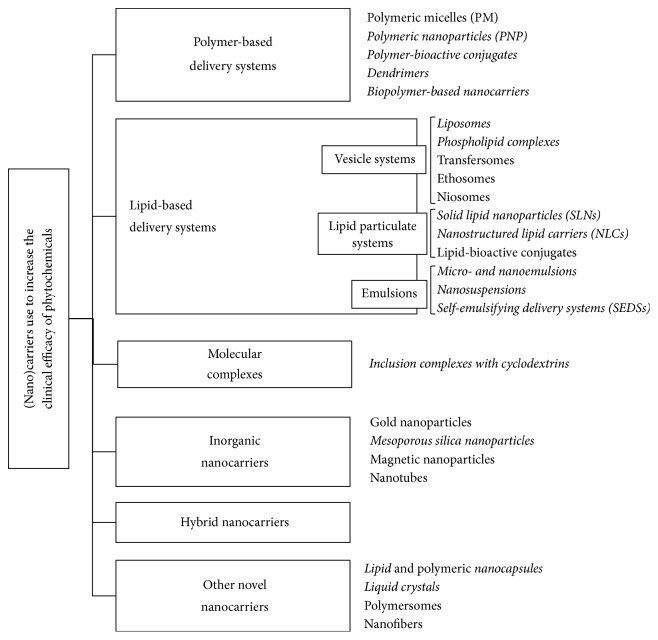
Types of (nano)carriers used to increase bioefficacy of phytochemicals. Those developed for oral administration of active compounds are in italic characters.

Over the last years, new formulation strategies to increase the clinical efficacy of poor water-soluble active compounds have been developed.  Figure 2 shows the new ones, specifically those developed for oral administration of active compounds (in italic). In addition, polymer-based delivery systems (PBDS) have also been popularly adopted to increase the clinical efficacy of some Phy, as observed in Table 4 [63, 64]. To a lesser extent, inclusion complexes with cyclodextrins and its derivatives as well as inorganic, hybrid, and other novel nanocarriers are being currently used (Table 4).

Furthermore, it is worth mentioning that, in recent years, an increased interest has been focused on the incorporation of poorly water-soluble compounds into* lipid-based delivery systems (LBDS)*. Association with lipid-based delivery systems has been shown to be one the most powerful strategies for the formulation of poorly water-soluble active compounds [8, 9], as they show several advantages compared to other carriers, including

- higher degree of biodegradability and biocompatibility;
- higher degree of versatility: lipid formulations can be modified in various ways to suit the stability requirements (molecular weight and physicochemical properties) and toxicity and efficacy of the active agent as well as the route of administration and cost;
- high and enhanced loading capacity;
- pharmaceutical stability;
- release of the active compound in controlled and targeted way;
- simple preparation methods and easy scale production;
- low risk of side effects (nontoxic).

The present work reviews the novel LBDS (vesicle and lipid particulate systems and emulsions) as recorded in Figure 2, describing the formulation approaches and mechanism of action. Furthermore, the LBDS combined with Phy* in vitro* and* in vivo* studies are also listed.

### 4.1. Formulation Approaches for Oral Lipid-Based Delivery Systems

LBDS can be obtained by blending excipients such as pure triglyceride oils, mixed glycerides, lipophilic surfactants, hydrophilic surfactants, and water-soluble cosolvents, which determine the absorption process [65]. Thus, in order to maximize the success in lipid-Phy formulation development and commercialization, it is precise to consider the following aspects:

- Screening and preselection of lipid excipients, mainly considering their solubility, dissolution/dispersion properties, digestibility, and absorption. Other factors are irritancy, toxicity, purity, chemical stability (regulatory issues), capsule compatibility, melting point (depending on the fatty acid composition), and cost
- Identification of the suitable formulation technique for the intended dosage form. Often solid form, developed mainly by adsorption on solid carriers [66], spray drying [67], lyophilization [68], melt extrusion [69], and nanoparticle technology [62], is preferred over liquid and semisolid forms, which offer low stability, irreversible drug/excipient precipitation, large volume of dose, and difficulty of handling and portability
- Testing the formulation in appropriate animal models to predict the* in vivo* behavior (bioavailability, pharmacokinetics, and intestinal lymphatic absorption)
- Optimization of the formulation based on the Phy loading and dissolution profile.

### 4.2. Mode of Action of Oral Lipid-Based Delivery Systems

**Figure 3:**
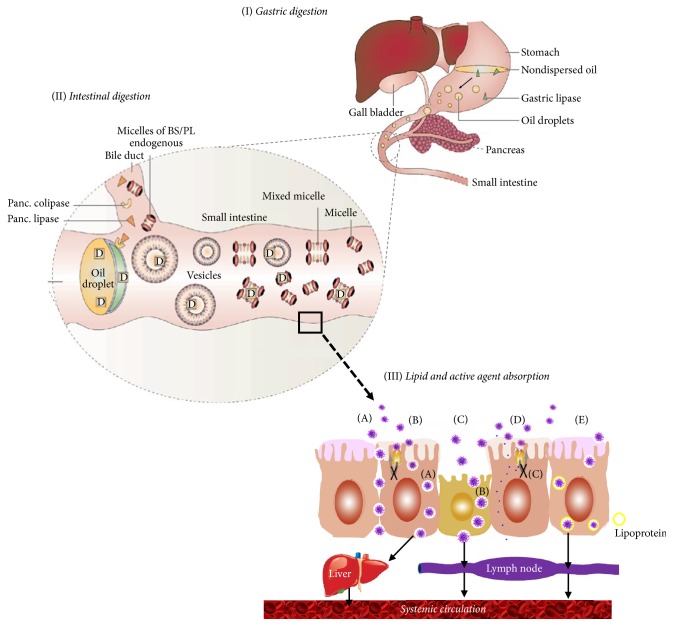
Mode of action of lipid-based delivery systems designed for the efficient oral administration of phytochemicals. (A) Allowing paracellular transport by opening tight junction; (B) facilitating transcellular absorption due to increased membrane fluidity; (C) promotion of phagocytosis via specialized microfold cells (M cells) of Peyer's patches; (D) increased intracellular concentration and residence time by surfactants due to inhibition of P-gp and/or CYP450; (E) lipid stimulation of lipoprotein/chylomicron production.

The goal of any oral LBDS is to enhance the GI absorption and oral bioavailability of the active compound. Their mode of action involves the alteration of the following physiological effects.

(I) After oral administration of the lipid-Phy formulation and once in the aqueous environment of the stomach, gastric lipase initiates the digestion of formulation lipids. Simultaneously, peristaltic movements of the stomach facilitate dispersion of lipid excipients into small droplets (Figure 3(I)). This accelerates the solubilization process of Phy in the lipid base and keeps the Phy in solution for prolonged period, avoiding its precipitation and protecting it from the low pH in stomach and the enzymatic and/or chemical degradation within the GI tract [1, 5, 6, 70].

(II) Once in the small intestine, lipid excipients stimulate bile flow and pancreatic juices excretion [71]. Pancreatic lipase hydrolyzes triglycerols (TG) into free fatty acids (FFA), monoglyceride (MG), and diglyceride (DG), which, along with bile salts and phospholipids (PL) from gallbladder, form* vesicles*, micelles, and mixed micelles (Figure 3(II)). These colloidal structures favor solubilization and transportation of Phy until absorption area protecting it from microbiota metabolism and enzymatic degradation, prolonging its residence time, and leading to the uniform distribution of Phy in the GI tract, which minimizes irritation of gut wall due to direct contact with Phy [1, 72].

(III) Formation of colloidal systems (vesicles, micelles, and mixed micelles) that significantly enhances the intestinal absorption of lipid digestion products and Phy as follows:

(i) Changing Phy uptake by interacting with transport processes of enterocyte. These include mucoadhesion (interaction with mucin to increase membrane fluidity), paracellular transport by modulating tight junctions, and promotion of receptor-mediated transport processes (endocytosis, transcytosis, and phagocytosis) via M cells of Peyer's patches and other mucosa-associated lymphoid tissues (MALT) (Figure 3(III)(A)–(C)).

(ii) Inhibiting efflux transporter P-glycoprotein (P-gp) and metabolism by cytochrome P450 (CYP450) or cytochrome 3A (CYP-3A) isozymes (Figure 3(III)(D)). This increases the intracellular concentration and residence time of Phy in enterocyte.

(iii) Enhancing Phy transport to the systemic circulation via intestinal lymphatic system [73–75]. Lipid metabolites stimulate lipoprotein/chylomicron production, which react with Phy molecules enhancing its intestinal lymphatic transport (Figure 3(III)(E)). This avoids the first-pass hepatic metabolism, which provides resistance to metabolic processes, leading to changes in Phy disposition and, finally, in its pharmacokinetic properties [70, 75].

All of this leads to an enhanced absorption, oral bioavailability, and bioefficacy of Phy, which should allow applying an accurate oral dosage to obtain reproducible results in clinical assays (reduced inter- and intrasubject variability) and enhance, thus, the clinical use of Phy therapy.

(IV) In addition of increasing water solubility, absorption, and oral bioavailability, lipid-based delivery systems have been shown to

- reduce the effect of coingested food on pharmacokinetics of the bioactive molecule [70];
- increase Phy pharmaceutical stability and lengthen its systemic circulation time [76];
- release Phy slowly over an extended duration (days or months) after a single administration (sustained release) [77];
- enhance penetration into tumoral matrices, promoting more reliable Phy access, and enhance blood-brain barrier permeability [78, 79];
- modulate the biodistribution of incorporated molecules, which leads to targeted effects and, hence, reduced side effects [1];
- overcome multidrug resistance [80];
- enhance efficiency of codelivery of active ingredients and therapeutic agents [81].

### 4.3. Types of the Main Oral Lipid-Based Delivery Systems and Their Applications

#### 4.3.1. Vesicle Systems

As indicated in Figure 2, lipid-based delivery systems can be classified in three categories, including vesicle systems, lipid particulate systems, and emulsions. Among the vesicle systems, liposomes and phospholipid complexes are the most frequently used.

*(i) Liposomes*. Liposomes are the most common and well-investigated nanocarriers for targeted drug/active delivery. The use of liposomes to deliver phytochemicals began in the 1980s as an approach to overcome limitations of clinical application of these compounds [1]. Conventional liposomes consist in small spherical vesicles, which present a simple bilayer membrane enclosing aqueous spaces. The lipids mainly used are phospholipids, so that, in an aqueous medium, the hydrophobic tails tend to gather together, while the hydrophilic heads are exposed towards water, thereby forming the round-shape vesicles. Amphiphilic nature of these systems makes them capable of encapsulating from hydrophilic agents, which can be located within the aqueous core, to hydrophobic substances, which can be embedded into the inner fatty acid layers [82–85].

Liposomes are highly biocompatible and possess self-assembly capacity. They are considered pharmacologically inactive with minimal toxicity [82–85], although they are not as immunologically inert as previously suggested [86]. Likewise, conventional liposomes have been shown to increase oral bioavailability and bioefficacy of loaded agents by

- improving their water solubility and stability;
- avoiding their early precipitation and intestinal and hepatic degradation;
- leading to drug concentration in tumoral tissues. This is because liposomes are preferentially delivered and passively accumulate here due to the high interstitial pressure, enhanced vascular permeability and retention, and the lack of functional lymphatic drainage of solid tumors (passive targeting effect) [87, 88];
- minimizing side effects.

However, conventional liposomes show some disadvantages that limit their applicability. These include poor stability in the systemic circulation and high recognition by reticuloendothelial system (RES), which leads to short circulation time (short shelf life) and low encapsulation efficacy expulsion of loaded molecules by intermembrane transfer [89].

**Figure 4:**
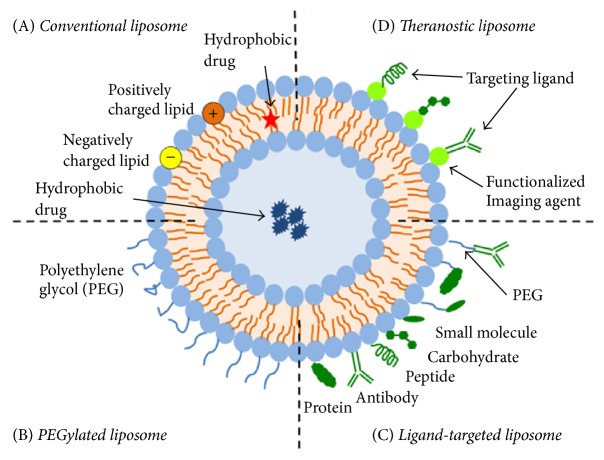
Schematic representation of the different types of liposomal drug delivery systems: (A) conventional liposome; (B) PEGylated liposome; (C) ligand-targeted liposome; (D) theranostic liposome (reprinted from Frontiers in Pharmacology, 6, article 286, 1–12. Advances and Challenges of Liposome Assisted Drug Delivery, by Sercombe et al. [87], with permission from the authors).

Over the last years, structural and physicochemical properties of liposomes have been modified to develop different types of liposomal delivery systems, called nanostructured liposomes, which do not show the drawbacks of the conventional ones [90] (Figure 4). Among them, we find the PEGylated liposomes, which are modified by adding polyethylene glycol (PEG) to the surface. This confers steric stabilization and, hence, higher stability* in vivo*. Structural modification can also consist in the attachment of different types of ligands (e.g., antibodies, peptides, and carbohydrates) to the surface or to the terminal end of the attached PEG chains. These systems, which are called ligand-targeted liposomes, are used for specific (active or physicochemical) targeting [91, 92]. Finally, to develop more efficient drug delivery systems, multifunctional liposomal formulations, also called theranostic liposomes, have been recently developed. These carriers usually consist of the nanoparticle, the therapeutic agent, an imaging component, and one or more targeting ligands which enhance their accumulation in pathological sites and promotes organelle-specific delivery. In this sense, theranostic liposomes can be used as therapeutic and diagnostic tool at the same time [87, 91].

The stability* in vitro* and* in vivo* of nanostructured liposomes as well as the release profile of the loaded agent is determined by the liposome surface charge, particle size, lipid composition, and number of lamellae and the nature of polymers and ligands attached to their surface [85, 93].

Nanostructured liposomes have been adopted in recent years for the efficient oral delivery of several Phy with poor water solubility and stability in the gastric environment (Table 5). Thus, for instance, vinorelbine, a chemotherapeutic obtained by semisynthesis from alkaloids extracted from the rosy periwinkle* (Catharanthus roseus)*, has been loaded into a cholesterol-polyethylene glycol (cho-PEG) coated liposome with the purpose of increasing circulating half-life and reducing severe side effects of this agent [94]. Likewise, N-trimethyl chitosan chloride- (TMC-) coated liposomes for the oral delivery of curcumin were found to be a promising strategy to reduce toxicity and increase therapeutic index [88].

Moreover, brucine, an alkaloid isolated from* Strychnos nux-vomica* L. (Loganiaceae), produced impressive dose-dependent antitumor effects by causing apoptosis. However, brucine was characterized by a narrow therapeutic index, and high doses of brucine cause severe central nervous system toxicity. Brucine-loaded stealth liposomes enhanced antitumor activity and decreased distribution to the brain [95], which, therefore, considerably improved its therapeutic index.

*(ii) Phospholipid-Phytochemical Complexes (Phytosomes®)*. Several plant bioactive compounds and extracts, mainly constituted by polyphenols and terpenoids, are conjugated with naturally occurring phospholipids, as phosphatidylcholine (PC), in a ratio of 1 : 1 or 1 : 2 (w : w). This formulation strategy leads to the formation of the patented complexes called Phytosomes. Like liposomes, structure of these complexes consists in spherical vesicles with a bilayer membrane of phospholipids, in which the hydrophilic heads are exposed towards the aqueous medium, while the hydrophobic tails remain together in the inner layer. Unlike liposomes, the active agent is not located within the aqueous core, but it binds to the polar end of phospholipid through weak chemical bonds, and the nonpolar portion of the phospholipid remains free [96, 97]. Phy-loaded phytosomes are highly biocompatible and bioavailable as compared to unloaded Phy. Incorporation into phytosomes increases the enterocyte cell membrane permeability of Phy and, hence, the amount reaching the systemic circulation. Likewise, phytosomes offer a controlled and sustained Phy release pattern, which leads to a longer action time and, therefore, to the need of a reduced Phy dose [96, 97].

Silybin was the first bioactive compound marketed as Phytosome formulation. Phospholipid complexation significantly increased the water solubility and liver protection of silybin, which resulted in an increase of its oral bioavailability and pharmacological activity [98]. In a comparative pharmacokinetic study using an equimolar dose of silybin and its complex, the plasma *C*_max_ of silybin after four hours was <35 ng/mL, whereas, for the silybin complex, it was 112 ng/mL [99]. Similarly, quercetin loaded-phytosome showed a water solubility 12-fold higher than free-form quercetin. However, complexation did not affect its antioxidant activity [100].

*Ginkgo biloba* L. and green tea extracts have been also loaded into phytosomes.* Ginkgo biloba* L. phytosome was supplied via oral to rats and, then, the pharmacokinetic profile of the major flavonoids of the extract (quercetin, kaempferol, and isorhamnetin) was evaluated by measuring their plasma *C*_max_, AUC_0_, and *T*_max_. Pharmacokinetic parameters of the three flavonoids were significantly improved after formulation, demonstrating that complexation with phospholipids leads to a large increase in Phy oral bioavailability [101]. Likewise, phytosome of green tea extract, principally represented by (−)-epigallocatechin 3-O-gallate, showed an enhanced absorption of catechins as compared to unloaded green tea catechins [102].

#### 4.3.2. Lipid Particulate Systems: SLNs and NLCs

Generally, there are two types of lipid nanoparticles (LNPs), solid lipid nanoparticles (SLNs) and nanostructured lipid carriers (NLCs) [103]. Both SLNs and NLCs have spherical shape and their average size usually ranges from 40 to 1000 nm. LNPs can be produced by several techniques such as high-pressure or high-speed homogenization, supercritical fluid extraction of emulsions, solvent emulsification/evaporation, spray drying, and ultrasonication [104–106].

**Figure 5:**
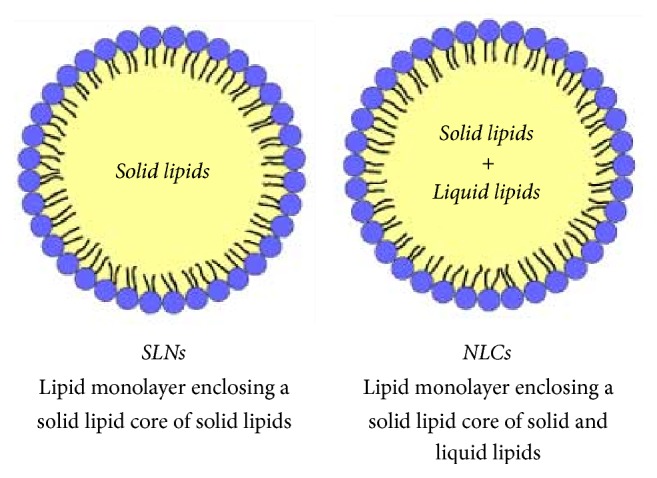
Structure of solid lipid nanoparticles (SLNs) versus nanostructured lipid carriers (NLCs).

LNPs are composed of a lipid solid matrix lipid and surfactants that provide stability [107]. SLN matrix is constituted by biocompatible, biodegradable, and GRAS solid lipids, which are solid at room and body temperature (e.g., highly purified triglycerides, partial glycerides, fatty acids, and steroids) [108]. The matrix of NCLs is also solid at room/body temperature; however, unlike SLNs, often it is composed of a mixture of solid and liquid lipids [101].  Figure 5 shows a scheme of these formulations, where structural differences between both LNPs are observed.

In the last years, a great attention has been paid to LNPs as an interesting and cost-effective alternative to polymeric nanoparticles, liposomes, and emulsions. LNPs are cheaper and safer than polymeric carriers, as their production is an organic solvent-free process [103]. Likewise, compared to conventional liposomes, nanoparticle solid matrix allows a higher control release and specific delivery of the loaded agent, which minimizes side effects [109]. LNPs show other benefits as compared to other systems, including ease of preparation and high scale production and sterilization [110, 111], excellent physical stability, and chemical versatility. Moreover, incorporation into the nanoparticle matrix can protect molecules from light, moisture, chemical degradation, and oxidation [109] and favor their penetration through mucus barrier due to nanosize [103, 112–114].

*(i) Solid Lipid Nanoparticles (SLNs)*. Despite all these advantages, applicability of SLNs presents several limitations such as the growth of matrix lipid particles, high water content, ease of gelation, and unpredictable polymorphic transitions, resulting in poor loading capacity [115–117]. In general, drug molecules stay in between the fatty acid chains or as amorphous clusters in crystal imperfections within SLN matrix. However, during SLN storage time, a transition of lipids to a low-energetic form can occur, giving rise to a perfect crystalline structure with very little space for the drug molecules. This promote the expulsion of encapsulated molecules, especially when SLN matrix is composed of a highly purified lipid, which results in a nanoparticle low incorporation capacity and a changing release profile with storage time [103, 113].

*(ii) Nanostructured Lipid Complexes (NLCs)*. To overcome SLNs drawbacks, NLCs have been developed as alternative carrier systems. The presence of liquid lipids (oil) in the solid matrix makes more imperfections to accommodate more active molecules than SLNs, which reduces the active molecule expulsion and enhances the nanoparticle loading capacity. Furthermore, the release and delivery of the active compound can be easily modulated by changing the lipid composition of matrix [113]. NLCs present a lower water content than SLNs and no significant differences regarding biotoxicity have been observed [118].

Table 5 shows* in vitro*/*in vivo* studies where SLNs and NLCs have been applied for the efficient oral delivery of antitumor Phy, mainly flavonoids, with limited therapeutic potential [119]. Thus, for instance, Luo et al. [120, 121] investigated the effect of loading puerarin, an isoflavonoid derived from* Radix Puerariae*, into SLNs, including pharmacokinetics, tissue distribution, and relative bioavailability in rats. When incorporated into the SLNs, puerarin was rapidly absorbed and its relative oral bioavailability was improved more than 3-fold as compared with that of the puerarin suspensions. In addition, SLNs produced increased tissue concentrations in puerarin target organs, particularly heart and brain. Likewise, triptolide, a diterpenoid epoxide isolated from* Tripterygium wilfordii* with anti-inflammatory, anticystogenesis, and anticancer effects, showed enhanced clinical efficacy and minimized side effects (irritation of the gastrointestinal tract) after encapsulation into SLN [122]. This was attributed to the solubilization of triptolide during GI digestion by the SLN matrix and colloidal mixed micelles (Figure 4), avoiding its precipitation and degradation as well as the GI irritation caused by insolubilized crystals. Moreover, SLNs minimize direct contact of triptolide with the mucosal surface and lead to a gradual release, avoiding high local and irritating concentrations.

Several Phy have been also loaded into NLCs in studies focused on improving water solubility, enhancing GI absorption and oral bioavailability, controlling release, increasing stability, and lengthening circulation time by reducing the recognition by the reticuloendothelial system (RES) (Table 5). The flavonoid silymarin has been used clinically to treat several hepatic disorders without a high efficiency. To improve oral absorption, silymarin-loaded NLCs were developed [123]. These formulations showed fast* in vitro* lipid digestion, suggesting that NLCs may facilitate the rapid silymarin absorption, and gave rise to relative silymarin bioavailability 2.54- and 3.10-fold greater than that produced by marketed LEGALON® and solid dispersion pellets, respectively. The ability of NLCs to enhance absorption was confirmed in other studies using tripterine, a triterpenoid from the Celastraceae family, extracted from the Chinese herbal plant* Tripterygium wilfordii* [124]. More recently, various novel and complex NLCs have emerged as carrier designed to achieve specific functions. For example, cell penetrating peptide- (CPP-) coated NLCs loaded with tripterine noticeably enhanced antitumor activity* in vitro* in prostate tumor cells, as well as in prostate tumor-bearing mice [124]. Ionic complex loaded NLCs enhanced the encapsulation efficiency, improved lipophilicity, and produced sustained release* in vivo* [95].

#### 4.3.3. Emulsions

*(i) Microemulsions and Nanoemulsions*. Microemulsions (MEs) are optically isotropic systems with special features, including an average particle size that ranges from 10 to 100 nm; spontaneous formation, that is, without any energy input; thermodynamic stability; optical transparence or slight opalescence; and low viscosity and allergenicity. All this makes them very attractive delivery systems [125].

MEs are constituted by an oil phase, an aqueous phase, a surfactant, and, probably, a cosurfactant [126]. When there are similar amounts of oil and water, a bicontinuous ME is usually formed, in which both phases form continuous domains separated by surfactant-stabilized interfaces. Otherwise, when amounts of oil and water are not similar, MEs with droplet-like structure are formed, which can be water-in-oil (w/o) or oil-in-water (o/w) MEs depending on the major compound.

Like other promising carriers, MEs have been shown to improve oral delivery of bioactive compound by (i) enhancing stability and permeability, (ii) allowing a controlled and sustained release, and (iii) improving GI absorption and oral bioavailability via the lymphatic transport pathway [1, 127]. In this respect, it has been found that this absorption pathway can be significantly favored by w/o MEs as compared to o/w MEs. In addition, due to their special features, MEs offer further advantages, such as ease of preparation, high capacity to solubilize hydrophilic, and lipophilic compounds and long-term stability.

Despite their numerous advantages, MEs present some limitations. They are sensitive to changes of environmental conditions, such as temperature, ionic strength, and composition (adding/removing molecules to/from the aqueous continuous phase), which may compromise their stability. In addition, MEs formation requires the use of relatively large amounts of synthetic surfactants to achieve an efficient loading capacity, especially when using triglycerides as dispersed oil phase [126].

Nanoemulsions (NEs), often also called miniemulsions, are systems with droplet-like structure. They are formed by an oil phase, an aqueous phase, and a mixture of surfactants and cosurfactants stabilizing droplets, whose average size is significantly (10-fold or so) smaller than that of droplets present in conventional emulsions [126]. Like MEs, they are optically transparent and show low viscosity. Moreover, although NEs do not form spontaneously and have been shown to be thermodynamically unstable, they show high kinetic stability, which can be for several years. As compared to MEs, these systems are much less sensitive to changes of environmental conditions and require lower amounts of synthetic surfactants to be formed due to their higher loading capacity [126].

Application of MEs and NEs as carriers for the efficient oral administration of Phy is shown in Table 5. Hydroxysafflor yellow A (HSYA) is a flavonoid derived and isolated from the safflower plant (*Carthamus tinctorius* L.) that has been shown to possess antioxidant and anti-inflammatory actions, antiplatelet aggregation, and antitumor properties as well as antimyocardial injury effects [128, 129]. Unlike other flavonoids, water solubility of HSYA is high; however, it has very poor permeability, which limits its GI absorption, oral bioavailability, and bioefficacy. Qi et al. [130] developed a HSYA-loaded ME (w/o), which showed a bioavailability ca. 19-folds higher than that of the unloaded compound. MEs have been also used to deliver poor water-soluble and stable Phy, such as elemenes (sesquiterpene). Elemene-loaded emulsions have been used clinically as antitumor agents. However, due to their poor stability and water solubility, the oral bioavailability of these emulsions was only 18.8%. An o/w elemene-loaded ME was then prepared [131]. This showed high entrapment efficiency of 99.81% and significantly higher stability than a normal emulsion, which led to a relative bioavailability 1.63-fold greater than that of the conventional emulsion (Table 5).

*(ii) Self-Emulsifying Delivery Systems*. A further and very successful approach to overcome problems associated with poor water solubility of Phy is self-emulsifying delivery systems (SEDSs), self-microemulsifying delivery systems (SMEDSs), and self-nanoemulsifying delivery systems (SNEDSs). These systems consist in isotropic mixtures, which include a large variety of liquid or waxy excipients available, ranging from oils through biological lipids (natural/synthetic oil) and hydrophobic and hydrophilic surfactants to water-soluble cosolvents, generally regarded as safe (GRAS) status [132]. Moreover, additives like *α*-tocopherol, *β*-carotene, and propyl gallate can be added to prevent the oxidation of SEDSs-Phy formulations [133].

Unlike all the previously described lipid formulations, these systems have a unique property: they remain in a preformulation state until ingestion. Upon dilution in aqueous physiological fluids of GI tract and with the gentle agitation provided by peristaltic movements, SEDSs are able to spread readily and self-emulsify spontaneously, forming fine o/w emulsions (50 nm > droplet size > 250 nm), that keep the active agent in solubilized form [134–136]. SEDDS formulations (oil, 40–80% (HLB < 12), 20–60%) commonly give rise to opaque dispersions with particle sizes >250 nm, while SMEDS formulations (oil, 40–80% (HLB > 11), 20–40%; hydrophilic cosolvents, 0–40%) disperse into smaller droplets with particle sizes between 50 and 250 nm, leading to optically clear or slightly opalescent microemulsions. SNEDS formulations (oil, <20% (HLB > 11), 20–50%; hydrophilic cosolvents, 20–50%) further disperse in GI fluids, giving rise to nanoemulsions with a droplet size less than 50 nm and completely transparent [9, 62].

The reduction in emulsion particle size of these formulations once in the GI tract increases the surface area of particles, which, in turn, provides higher interfacial surface area and a very low interfacial tension. This provides SEDSs with a high capacity to solubilize the loaded Phy in the GI tract and to enhance its release and absorption and oral bioavailability [136–138]. It should be noted that droplet size of o/w emulsions formed after self-emulsification inside the body and, hence, capacity of SEDSs to act as efficient Phy carriers is highly determined by the excipient combination used in the formulation of these systems. Therefore, selection of excipients is a quite challenging task that should be considered.

Besides improving oral bioavailability of poor water-soluble Phy, SEDSs show multiple advantages. Among them are the following:

- Formulation surfactants increasing the intestinal permeability, which decreases surface tension and facilitates formulation contact with intestinal mucus [139]
- SEDSs protecting loaded Phy against enzymatic degradation and avoid its first-pass hepatic metabolism
- SEDSs providing higher loading capacity than conventional lipid solutions
- Thermodynamic stability
- Ease of manufacture and scale-up. These advantages make SEDS unique when compared to other drug delivery systems like solid dispersions, liposomes, nanoparticles, and so forth [140–142]
- Ease of administration and versatility of dosage form, in either liquid or solid form. Liquid dosage forms can be administered in soft or hard gelatin capsules but these have shown some drawbacks, such as high production costs, low drug compatibility and stability, drug leakage and precipitation, capsule ageing, and need of a large quantity of surfactants (30–60%), which can induce GI irritation. These disadvantages are overcome by formulating SEDS as solid forms by extrusion/spheronization methods [72].

The delivery of poorly water-soluble Phy using SEDSs has been extensively studied during the past decade and many of these studies are summarized in Table 5. Thus, for instance, the self-double emulsifying formulation of Hydroxysafflor yellow A (HSYA) was developed using phospholipid dissolved in Labrafac™, Lipophile WL1349, Tween 80, and oleic acid. The formulation results in 20-fold increase in *C*_max_ and 35-fold rise in AUC value of Phy as compared to the aqueous solution [143, 144]. The SMEDS of gentiopicrin obtained from the roots of gentians was formulated using phospholipids in Labrasol as oil phase and Cremophor EL and Transcutol P as other excipients. The SMEDS of gentiopicrin with phospholipids enhanced the relative bioavailability of Phy to 703.62% as compared to gentiopicrin alone. Similarly, the phospholipid complex of morin (MPC) was developed as SNEDS using Labrafil M1944 CS, Cremophor RH 40, and Transcutol P as excipients which exhibited a significant increase in *C*_max_, *T*_max_, and relative oral bioavailability (6.23-fold) as compared to the morin suspension [145]. Likewise, lutein formulated as SNEDDS demonstrated having immediate dissolution (within 5 min) as compared to commercial product of lutein (Eyelac®) where there is no dissolution within specific time [146]. Many other studies have been carried out to enhance oral bioavailability and therapeutic effect of other plant active compounds, including apigenin, berberine hydrochloride (BBH), puerarin, hesperidin, quercetin, curcumin, baicalin, oleanolic acid, vinpocetine, nobiletin, oridonin, and silymarin.

## 5. Other Approaches to Increase Bioefficacy of Antitumor Phytochemicals

### 5.1. Oral Codelivery of Phytochemicals and Chemotherapeutic Drugs

Combined cancer therapy consisting in (i) the combined application of some of the most common types of cancer treatment, including surgery, radiotherapy, chemotherapy, targeted therapy, and immunotherapy or (ii) the coadministration of different chemotherapy drugs, is often more effective. The rationale for combination chemotherapy is to use drugs that work by different mechanisms, thereby decreasing the likelihood that resistant cancer cells will develop. Moreover, when drugs with different effects are combined, each drug can be used at its optimal dose, without intolerable side effects [147].

Following the same rationale, it is believed that codelivery of antitumor drugs and plant bioactive compounds could improve therapeutic effects by targeting diverse molecular targets, reducing toxicity, overcoming drug resistance, and facilitating the use of lower and safer doses [1]. Thus, as observed in Table 3, there are many* in vitro* and* in vivo* studies as well as some clinical trials focused on demonstrating the potential synergistic effect when codelivering phytochemicals, mainly polyphenols, and first line chemotherapeutic agents [148, 149].

Codelivery strategy is, however, usually limited by low water solubility, poor oral bioavailability, undesirable pharmacokinetic characteristics, and side effects [1]. In this sense, incorporation of two or more molecules (Phy + Phy or Phy + drug) in one nanocarrier seems to be a promising way to increase the bioefficacy of codelivery method. It has demonstrated to (i) improve water solubility and oral bioavailability; (ii) suppress drug resistance, by inhibiting transporter mediated efflux; (iii) delay adaptation processes; (iv) retard cells mutations; (v) produce synergistic therapeutic effect through the simultaneous delivery of multiple agents to the action site; and (vi) minimize side effects [1, 150].

In this sense, few Phy described in Table 3 have been coencapsulated or coloaded in one oral nanocarrier. Quercetin + tamoxifen was administrated through PLGA nanoparticles, while quercetin + paclitaxel was administrated through CQ-PM and curcumin + genistein through NLC.

### 5.2. Parenteral and Topical Administration of Phytochemicals as Alternative to the Oral Route

To overcome limitations in the oral administration of poor water-soluble Phy, parental (intravenous and intraperitoneal) and topical (transdermal, nasal, and ocular) administration routes can be used to increase dose precision and clinical efficacy.

Likewise, in recent years, topical delivery of bioactive compounds has also drawn great attention owing to its advantages over other administration routes and outstanding contribution in improving local action [151] or systemic absorption, which can minimize the first-pass effect [152]. Nevertheless, this application also shows several barriers that limit its use, including low skin permeation, short biological half-life, presystemic metabolism, or systemic toxicity [1].

On the other hand, and to get over limitations of parenteral and topical administration routes, application of nanocarriers has demonstrated to be also an efficient formulation strategy.  Table 6 shows and overviews the lipid and nonlipid formulations specifically designed to parenteral and topical Phy administration. In case of the parenteral route, most of the investigations have focused on utilizing carriers to enhance antitumor efficiency through passive targeting or active targeting [153, 154], controlling drug release at the tumor site to minimize side effects [155, 156], or overcoming multidrug resistance [157]. Parenteral nanocarriers include either lipid formulations (liposomes, SLNs, and NCLs) or polymer formulations (polymeric NPs and polymer-bioactive conjugates). For topical application, the incorporation of active compounds into nanocarriers aims to enhance skin permeation and stability, lengthen systemic circulating time, and minimize metabolic degradation and systemic toxicity. Thus, for instance, MEs provide a safe, effective, and noninvasive means to topically deliver Phy such as quercetin [158], genistein [159], and chlorogenic acid and resveratrol [160]. Other nanocarriers used for the topical delivery of Phy include liposomes, ethosomes, NLCs, polymeric NPs, and polymer-bioactive conjugates (Table 6).

## 6. Conclusions

Phy are molecules obtained from natural plant species and in the last decades have shown their positive benefits in human health, in prevention and treatment.

In the framework of cancer, polyphenols are the most studied group of phytochemicals, in both the* in vitro*/*in vivo* studies and clinical trials, with promising expectative, including the lack of side effects. Regarding terpenes, phytosterols, and organosulfur phytochemicals, they show hopeful results in breast, colon, and prostate models, although there are few clinical trials that started to confirm their effects in human models, compared with polyphenols.

The bioavailability of these compounds still adheres to measure urine levels as a routine parameter, but many authors defend the use of carriers to improve their availability in plasma and in targeted organs. This need is reflected in the development of new delivery mechanisms, where lipid-based delivery systems are part of a strategy to increase the water solubility and stability, prevent the rapid systemic clearance, prevent the intestinal and hepatic metabolism, enhance the bioavailability, and enhance the cancer cell targeting. The importance of measuring tissue levels of the chemopreventive agents would help to better understand the mode of action of the nanoparticles and phytochemicals and to avoid toxicity of both.

## Figures and Tables

**Table 1:** Polyphenols studied in experimental *in vitro* tests, *in vivo* models, and clinical trials.

Polyphenols	Phytochemical	Main source	Cancer targets *in vivo* and *in vitro*	Clinical trials	References cancer targets/clinical trials	Chemical structure
Phenolic acids	Ellagic acid	Pomegranate, berries, grapes	*Prostate*	Prostate Follicular lymphoma	[161–165]/ [166, 167]	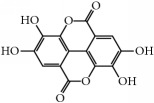
			*Pancreas*			
			Bladder			
			Breast			
			Colon			

Flavonoids	(−)-Epigallocatechin-3-gallate (EGCG)	Green tea *(Camellia sinensis)*	*Prostate*	Prostate Papilloma cervical Breast Prostate	[168–174]/ [175–178]	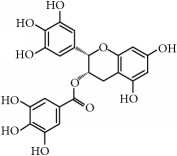
			*Renal carcinoma*			
			*Breast*			
			Laryngeal carcinoma			
			Non-small cell lung			
			Colon			
			Pancreas			
	Genistein	Soybean	*Bone marrow*	Prostate	[179–183]/ [184–187]	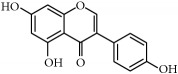
			*Prostate*	Bone		
			Breast	Endometrial		
			Cervical	Breast		
			Colon	Bladder		
	Luteolin	Cabbages, celery, broccoli, onion leaves, parsley	*Hepatocellular carcinoma*	—	[188–193]	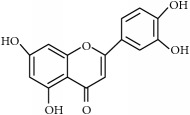
			*Oral squamous carcinoma*			
			Prostate			
			Breast			
			Thyroid			
			Colorectal			
			Cervical			
			Lung			
	Silymarin	Thistle *(Silybum marianum)*	*Prostate*	Upper gastrointestinal Leukemia	[194–198]/ [199, 200]	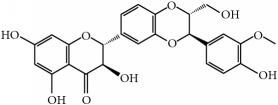
			Breast			
			*Ovary*			
			Colon			
			Lung			
			Bladder			
			*Skin*			
			Prostate			
	Quercetin	Capers, lovage leaves, apple	Pancreas	Large bowel	[201–206]/ [207–209]	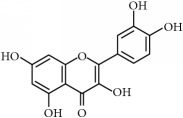
			*Breast*	Ovary		
			Cervical	Pancreas		
			Colon	Prostate		
			Prostate	Thrombotic		
			*Lung*	Colorectal		

Stilbenes	Resveratrol	Grape, berries	*Breast*		[38, 39, 210–217]/ [218–222]	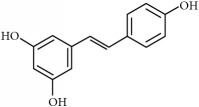
			*Colorectal*			
			*Hepatic melanoma*			
			*Lung*	Colorectal		
			*Pancreas*	Colon		
			*Prostate*	Gastrointestinal tumors		
			*Skin*			
			*Bladder*			
			*Ovarian*			

Curcuminoids	Curcumin	*Curcuma longa* L.	*Pancreas*	Pancreas Colorectal Colon Liver Pancreas Breast Head and neck	[40, 41, 223–230]/ [96, 231–238]	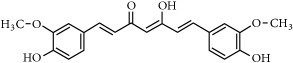
			*Prostate*			
			*Ovarian*			
			*Melanoma*			
			*Head and neck squamous cell carcinoma*			
			*Leukemia*			
			*Hepatoma*			
			*Gastric*			
			*Glioblastoma*			
			*Lung*			
			Breast			
			Cervical			
			Colorectal			

Clinical trials carried out considering phytochemicals as dietary complements or drugs (therapy) in cancer patients.

For the experimental studies, *in vivo* studies are in italic characters.

Chemical structures were obtained by using ChemDraw Professional 15.0 software.

**Table 2:** Terpenes, organosulfur, and phytosterols commonly studied in cancer therapy.

Family	Phytochemical	Main source	Cancer targets *in vivo* and *in vitro*	Clinical trials	References cancer targets/clinical trials	Chemical structure
Terpenes						

Carotenoids	Lycopene (tetraterpene)	Tomato *(Lycopersicon esculentum)*	*Prostate*	Prostate	[239–242]/ [243, 244]	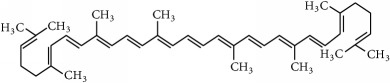
			*Colon*			
			Breast			
			Lung			
			Cervical			
			Breast			
			Laryngeal			
			Liver carcinoma			
	Astaxanthin	Green microalgae *(Haematococcus pluvialis)*	*Hepatic*	—	[245–251]	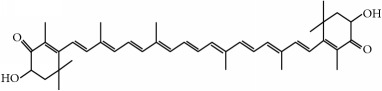
			*Oral carcinoma*			
			*Fibrosarcoma*			
			*Skin*			
			*Bladder*			
			*Colon*			
	*β*-Elemene	Ginger, celery	*Laryngeal*	Glioma	[252–256]/ [257]	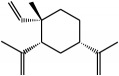
			Non-small cell lung			
			Gastric cancer			
			Prostate			
			Brain			
			Breast			
			Cervical			
			Colon			
			Ovarian			
			Melanoma			
			Glioblastoma			
Noncarotenoid	Carnosol (diterpene)	Sage *(Salvia carnosa)*, Rosemary *(Rosmarinus officinalis)*	*Colon*	—	[258–263]	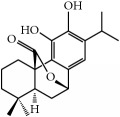
			*Prostate*			
			*Skin*			
			Breast			
			Ovarian			
			Intestinal			
			Melanoma			

Organosulfur						

Thiosulfinates	Sulforaphane	Brassica vegetables	*Skin*	Breast	[42, 95, 264–267]/ [268]	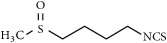
			*Gastrointestinal-colon*			
			*Prostate*			
			*Pancreas*			
			*Breast*			
			*Bladder*			
			Ovary			
			Mammary			
	Diallyl disulfide	Allyl vegetables	*Gastric*	—	[43, 44, 269–273]	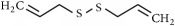
			*Breast*			
			*Leukemia*			
			Neuroblastoma			
			Prostate			
			Colon			
			Thyroid			

Phytosterols						

Phytosterols	*β*-Sitosterol	Vegetal oils	*Colon*	—	[274–278]	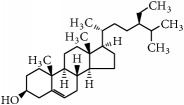
			Breast			
			Stomach			
			Prostate			
			Fibrosarcoma			

Clinical trials carried out considering phytochemicals as dietary complements or drugs (therapy) in cancer patients.

For the experimental studies, *in vivo* studies are in italic characters.

Chemical structures were obtained by using ChemDraw Professional 15.0 software.

**Table 3:** Phytochemicals combined with first-line antitumor drugs and their study in clinical trials. Nanocarriers used to enhance bioefficacy of codelivery are also shown.

Phytochemical	Codelivered antitumor agent	*In vitro*/*in vivo*	Clinical trial	Phase of study	Ref.
Ellagic acid	5-Fluorouracil	Colon	—	—	[279]
	Vinorelbine	—	Hormone refractory prostate cancer	Completed	[167]
	*α*-Difluoromethylornithine	Colon	—	—	[280]

(−)-Epigallocatechin-3-gallate (EGCG)	Tamoxifen + sulindac	Lung	—	—	[281]
	Sulindac	Intestinal	—	—	[281]

Genistein	Tamoxifen	Breast	—	—	[282]
	Gemcitabine hydrochloride	Pancreas	Breast	Completed	[283–285]
		Osteosarcoma			
	Decitabine	—	Pediatric solid tumors, leukemia	Recruiting	[286]
	Decitabine	—	Non-small cell lung	Completed	[287]
	Interleukin-2 (high-dose)	—	Kidney cancer	Completed	[288]
			Melanoma		
	5-Fluorouracil	Colon	—	—	[289]
	Docetaxel	Prostate	—	—	[290]
		Lung			
		Breast			
		Pancreas			
	Doxorubicin	Prostate	—	—	[290]
		Lung			
		Breast			
		Pancreas			
	Cisplatin	Ovarian	—	—	[290, 291]
		Prostate			
		Lung			
		Breast			
		Pancreas			
	Erlotinib	—	Pancreas	Completed	[292]
	Erlotinib + gemcitabine	Pancreas	Pancreas	Completed	[293, 294]

Luteolin	Celecoxib	Breast	—	—	[295]

Quercetin	Docetaxel	Prostate	—	—	[296]
	5-Fluorouracil	Esophageal	—	—	[297–299]
		Colorectal			
		Liver			
	Sulindac	Colorectal	Colon	Completed	[300]
	Tamoxifen	Breast	—	—	[301]
	Paclitaxel	Liver	—	—	[302]

Resveratrol	Rapamycin	Breast	—	—	[303]
	Doxorubicin	Breast	—	—	[304]
	Temozolomide	Glioma	—	—	[305]
	5-Fluorouracil	Colon			[306]
	Mitomycin	Colorectal	—	—	[307]

Curcumin	Irinotecan	Colorectal	Colorectal	Active	[308, 309]
	Folfox		Colon	Active	[310]
	Sulindac	Lung	Colorectal	Completed	[224, 311]
	Capecitabine		Rectal	Active	[311]
	5-Fluorouracil	Colorectal	—	—	[312]
	Dasatinib	Colon	—	—	[313]
	Paclitaxel	Breast			[314]
	Celecoxib	Colon			[315]
	Gemcitabine	Lung	—	—	[316]
	Genistein	Prostate	—	—	[317]

Lycopene	Docetaxel	Prostate	Adenocarcinoma of the prostate	Active	[318, 319]

**Table 4:** Overview of nonlipid formulations, which have been designed to administer phytochemicals by oral route.

Active ingredient	Lipid-based formulation		Effect of formulation	Ref.
	Type	Subcategory		
Curcumin	PBDS	PLGA^a^-NPs	Overcome multidrug resistance and increased oral bioavailability *in vivo*.	[320]
Silymarin			*In vitro* sustained release and enhanced cytotoxicity.	[321]
Curcumin		Hydroxypropyl cellulose NPs	Temperature-dependent release *in vitro*.	[322]
Puerarin		Dendrimers	Increased *in vitro* oral bioavailability and reduced side effects.	[323, 324]
Curcumin				
Resveratrol				
Genistein				
Podophyllotoxin				
Curcumin		Hyaluronic acid conjugate	Improved water solubility, stability, and antitumoral activity *in vitro*.	[325]
		Alginate conjugate	Higher water solubility, stability, and cytotoxicity *in vitro*.	[326]

Rutin	CD inclusion complexes	*α*-CD, *β*-CD, HP-*β*-CD, and DM-*β*-CD^b^	Improved water solubility and stability, increasing the oral bioavailability and bioefficacy.	[327]
3-EGCG				[328]

Silymarin *(Silybum marianum)*	Inorganic nanocarriers	Porous silica nanoparticles (PSN)	Sustained release and enhanced oral bioavailability *in vivo*.	[321]
Silybin meglumine				[329]

Resveratrol	Hybrid nanocarriers	TCC^c^- liposomes	Improved absorption and oral bioavailability and reduced side effects *in vivo In vitro* controlled release and *in vivo* enhanced targeting and reduced side effects Overcome multidrug resistance. Enhanced *in vitro* and *in vivo* antitumor activity.	[330]
		DQA-PEG_1930_-DSPE^a^ liposomes		[153]
Vincristine		Dextran-sulfate-SLNs		[331]
		PLGA-PEG-R7^a^ NPs		[332]
Tripterine		CPP^a^-NLCs		[118]

Silymarin	Other novel nanocarriers	Liquid crystalline nanocarrier	Sustained release. Improved water solubility, oral bioavailability, and biological activity (active targeting-liver) *in vivo*.	[333]
Quercetin		Folate-modified lipid nanocapsules		[334]
Tetrandrine		Lipid nanocapsules		[335]

^a^PLGA: poly(lactic-co-glycolic acid); PEG: polyethylene glycol; R7 is a cell-penetrating peptide; DQA: dequalinium; DSPE: polyethylene glycol-distearoylphosphatidylethanolamine; R7 is a cell-penetrating peptide (CPP).

^b^ *α*/*β*-CD: alpha/beta-cyclodextrin; HP-*β*-CD: hydroxypropyl-*β*-cyclodextrin; DM-*β*-CD: dimethyl-*β*-cyclodextrin.

^c^TCC: N-trimethyl chitosan chloride-coated.

**Table 5:** Overview of lipid-based delivery systems to administer phytochemicals by oral route.

Active ingredient	Lipid-based formulation	Effect of formulation	Ref.
Vinorelbine	Liposomes	Reduced side effects and increased circulation half-life.	[94]
		Improved therapeutic effect *in vivo*.	
Gypenoside		Activated *in vitro* immune response in macrophages.	[335]
Curcumin		Improved pharmacokinetics and oral bioavailability *in vivo*.	[329, 336]
3-EGCG		Enhanced *in vitro* antitumor activity.	[337]
Brucine		Improved absorption and oral bioavailability, enhanced targeting, and reduced side effects *in vivo*.	[338, 339]

Quercetin	Phytosome	Enhanced membrane permeability, sustained and controlled release. Enhanced absorption, oral bioavailability, and bioefficacy.	
Kaempferol			[101]
Isorhamnetin			
Silybin			[98, 99]
3-EGCG			[102]
Quercetin			[100]

*β*-Elemene	Microemulsions	Increased water solubility and permeability and improved oral bioavailability.	[131]
Hydroxysafflor yellow A			[130]
Puerarin			[127, 340]

Baicalin	SEDS	Enhanced stability, oral bioavailability, and targeting effects *in vitro* and *in vivo*.	[134]

Curcumin	SMEDS	Enhanced stability, oral bioavailability, and targeting effects *in vitro* and *in vivo*.	[341]
Indirubin			[88]
Hydroxysafflor yellow A			[143, 144]
Gentiopicrin			[341]
Lutein			[342]
Apigenin			[343]
Nobiletin			[137]
Oridonin			[139]
Silymarin			[140]
Puerarin			[344]
Hesperidin			[345]
Berberine hydrochloride (BBH)			[346]

Morin	SNEDS	Enhanced stability, oral bioavailability, and targeting effects *in vitro* and *in vivo*.	[145]
Curcumin			[347]
Lutein			[146]
Oleanolic acid			[348]
Vinpocetine			[349]

Puerarin	SLNs	Improved absorption and oral bioavailability and reduced side effects (irritation of GI mucous membrane) *in vivo*.	[120, 121]
Triptolide			[350]
Cantharidin			[351]
Resveratrol			[352]

Silymarin	NLCs	Increased absorption and oral bioavailability *in vivo*. Enhanced *in vitro* and *in vivo* antitumor activity.	[123]
Tripterine			[124]
Curcumin			

**Table 6:** Overview of lipid and nonlipid formulations, which have been designed to administer phytochemicals by parental and topical routes.

Phytochemical	Lipid-based formulation		Effect of formulation	Admin. route	Ref.
	Type	Subcategory			
Curcumin	LBDS	NLCs	Enhanced stability and brain targeting *in vivo*.	Intraperitoneal	[353]
Baicalein	LBDS	Tocol-NLCs			[354]
*β*-Elemene		NLCs	Less irritating and toxic and enhanced bioavailability and antitumor efficacy *in vivo*.		[355]
Bufadienolides			Reduced toxicity and improved pharmacokinetic profile *in vivo*.	Intravenous	[356]
Breviscapine		Ionic-complex-based NLCs	Sustained-release and protection against liver enzyme degradation *in vivo*.		[357]
Berberine		DQA-PEG_2000_-DSPE^a^ liposomes	Overcome multidrug resistance *in vivo*.		[358]

Quercetin	LBDS	MEs		Transdermal	[158]
Genistein			Increased permeation and skin retention.		[159]
Chlorogenic acid			Efficient systemic distribution *in vivo*.		[160]
Resveratrol					
Curcumin		PEG^a^ liposomes	Increased stability and anti-inflammatory effects *in vivo*		[359]
Bufadienolides		Poloxamer-liposomes	Reduced toxicity and enhanced antitumor efficacy *in vivo*.		[360]
Ligustrazine phosp.		Ethosomes	Enhanced skin permeation *in vitro* and bioactivity *in vivo*.		[152]
Apigenin			Enhanced anti-inflammatory effects *in vivo*.		[361]
Curcumin		NLCs	Enhanced antitumor activity and brain targeting *in vitro*.	Intranasal	[362]
Tetrandrine		Charged SLNs	Reduced irritation of eye mucous membrane in vivo.	Ocular	[363]

3-ECGC	Inorganic carriers	Gold NPs	Enhanced efficacy and reduced toxicity *in vivo*.	Intratumoral injection	[155]

Curcumin	PBDS	Dextran sulfate-chitosan NPs	Controlled release and targeted effect against tumor cells *in vitro*.	Intravenous	[364]
Curcumin		Chitosan/PBCA^b^ NPs	*In vivo* anticancer effect on hepatic tumor cells.		[365]
Trans-resveratrol		Chitosan-NPs	Higher *in vivo* liver targeting effect and *in vitro* cytotoxicity on hepatic cancer cells.		[366–368]
Oridonin		Galactosylated chitosan NPs	Enhanced targeting and binding to the specific site of action (liver).		

Artemisinin	PBDS	Polymeric micelles Targeted polymeric micelles	Achieving site-specific cell targeting and enhancing intracellular drug accumulation.	Intraperitoneal	[369]
Resveratrol		Transferrin modified PEG-PLA^c^ conjugate	Cellular uptake, *in vivo* biodistribution, and antitumor activity. Targeted therapy of glioma.		[370]
Bufalin		Biotinylated chitosan NPs	Enhanced targeting and binding to the specific site of action breast carcinoma.		[371]

Quercetin	PBDS	Lecithin-chitosan NPs	*In vitro* and *in vivo* enhanced skin permeation.	Topical	[371]

^a^PEG: polyethylene glycol; DQA: dequalinium; DSPE: polyethylene glycol-distearoylphosphatidylethanolamine.

^b^PBCA: poly(butyl cyanoacrylate).

^c^PLA: polylactic acid.
